# Fluorescent marker prediction for non-invasive optical imaging in bovine satellite cells using deep learning

**DOI:** 10.3389/frai.2025.1577027

**Published:** 2025-09-26

**Authors:** Sania Sinha, Aarham Wasit, Won Seob Kim, Jongkyoo Kim, Jiyoon Yi

**Affiliations:** ^1^Department of Computer Science and Engineering, Michigan State University, East Lansing, MI, United States; ^2^Department of Animal Science, Michigan State University, East Lansing, MI, United States; ^3^Department of Biosystems and Agricultural Engineering, Michigan State University, East Lansing, MI, United States

**Keywords:** deep learning, fluorescence, digital staining, non-invasive optical imaging, tissue biology, bovine satellite cells

## Abstract

Assessing the quality of bovine satellite cells (BSCs) is vital for advancing tissue engineered muscle constructs with applications in sustainable protein research. In this study, we present a non-invasive deep learning approach for optical imaging that predicts fluorescent markers directly from brightfield microscopy images of BSC cultures. Using a convolutional neural network based on the U-Net architecture, our method simultaneously predicts two key fluorescent signals, specifically DAPI and Pax7, which serve as biomarkers for cell abundance and differentiation status. An image preprocessing pipeline featuring fluorescent signal denoising was implemented to enhance prediction performance and consistency. A dataset comprising 48 biological replicates was evaluated using statistical metrics such as the Pearson *r* (correlation coefficient), the mean squared error (MSE), and the structural similarity Index (SSIM). For DAPI, denoising improved the Pearson *r* from 0.065 to 0.212 and SSIM from 0.047 to 0.761 (with MSE increasing from 9.507 to 41.571). For Pax7, the Pearson *r* increased from 0.020 to 0.124 and MSE decreased from 44.753 to 18.793, while SSIM remained low, reflecting inherent biological heterogeneity. Furthermore, enhanced visualization techniques, including color mapping and image overlay, improved the interpretability of the predicted outputs. These findings underscore the importance of optimized data preprocessing and demonstrate the potential of AI to advance non-invasive optical imaging for cellular quality assessment in tissue biology. This work also contributes to the broader integration of machine learning and computer vision methods in biological and agricultural applications.

## 1 Introduction

Advancements in technology are crucial for accelerating and automating the assessment of source cell quality in tissue engineered muscle constructs designed for sustainable protein production. Bovine satellite cells (BSCs), isolated from animal muscle tissue, are essential for developing muscle tissues due to their ability to proliferate and differentiate into skeletal muscle cells, driving tissue formation in engineered systems. Ensuring efficient proliferation and differentiation of these cells is essential for producing high-quality constructs that can serve as alternatives to conventional protein sources (Messmer et al., 2022; Stout et al., 2023). Traditionally, evaluation methods have relied on immunofluorescence microscopy (Lee et al., 2021; Kong et al., 2023). While microscopy provides valuable insight into cell morphology, its limited contrast and specificity for complex samples often necessitate additional fluorescent dyes or antibodies. These techniques require invasive sample preparation and expert annotation. Variability in cell isolation and culture conditions can affect the metabolic state and cellular composition, further impacting the binding efficiency and specificity of fluorescent stains (Kong et al., 2023). This underscores the need for non-invasive optical imaging methods to assess cell quality, especially considering the heterogeneity present during cell proliferation and differentiation.

Recent advances in artificial intelligence (AI) have enabled the automation of cellular image analysis. These efforts include deep learning segmentation of subcellular components to reduce the burden of expert annotation (Kromp et al., 2021; Bilodeau et al., 2022). Furthermore, predicting fluorescent signals from more cost-effective brightfield microscopy images can minimize the need for invasive staining (Christiansen et al., 2018; Ounkomol et al., 2018; Cheng et al., 2021). In 2018, Google first introduced *in silico* labeling, a deep learning approach that predicts fluorescent signals from transmitted light z-stack images of unlabeled samples (Christiansen et al., 2018). Additionally, convolutional neural network (CNN) models based on the U-Net architecture (Ronneberger et al., 2015) have demonstrated the ability to predict fluorescent signals for individual subcellular components, such as DNA, cell membranes, and mitochondria, directly from transmitted (Ounkomol et al., 2018) and reflective (Cheng et al., 2021) light microscopy brightfield z-stack images. These studies highlight the potential of AI-enabled image analysis to bridge the gap between traditional and digital techniques, suggesting a promising direction for improving non-invasive optical imaging for cellular assessment. Yet, these applications have predominantly focused on the biomedical sector, where cells are well-characterized and homogeneous (e.g., continuously proliferating human cancer cell lines), unlike the structurally variable BSCs that require advanced methods for precise assessment.

To address the complexity and variability inherent in BSC differentiation, it is crucial to incorporate enhanced visualization techniques into the analysis pipeline. These techniques improve interpretability and explainability, making it easier for researchers to understand model predictions. Recent advancements have demonstrated how improved visualization methods can be applied to biological image analysis, providing clearer insights and improving the reliability of AI predictions (Samek et al., 2017). Applying these techniques in predicting fluorescent or colorimetric signals has shown promise in bridging the gap between traditional and digital techniques (Binder et al., 2021; Cho et al., 2022). Therefore, integrating enhanced visualization methods is essential for advancing non-invasive techniques in the assessment of BSC quality, ultimately supporting the development of reliable and actionable AI-driven assessments.

In this study, we demonstrate a non-invasive optical imaging approach for quality assessment of cell culture isolated from bovine muscle tissues, employing deep learning to predict fluorescent signals from brightfield microscopy images. Specifically, we used two key biomarkers to determine the abundance of BSCs in the isolated cell culture, i.e., 4′,6-diamidino-2-phenylindole (DAPI) and paired box protein 7 (Pax7). DAPI is a widely used fluorescent stain that binds to cell DNA in fixed cells and tissues, while Pax7 serves as a transcription factor regulating the development and maintenance of skeletal muscle tissue, recognized as the most specific marker of satellite cells (Seale et al., 2000; Ding et al., 2018). Consequently, co-staining of cell cultures with DAPI and Pax7 is commonly utilized to monitor the proliferation and differentiation abilities of satellite cells over time (Ding et al., 2018; von Maltzahn et al., 2013). We employed a deep CNN based on the U-Net architecture, adapted from a previous study (Ounkomol et al., 2018) with a modified image preprocessing pipeline. The model architecture was trained on our microscopy images to predict multiple fluorescent markers from a single brightfield image of isolated BSCs. Overall, this deep learning approach provides digital staining by learning the features of subcellular components without invasive sample preparation, thereby accelerating cell quality assessment and reducing resource demands.

## 2 Method

**Figure 1:**
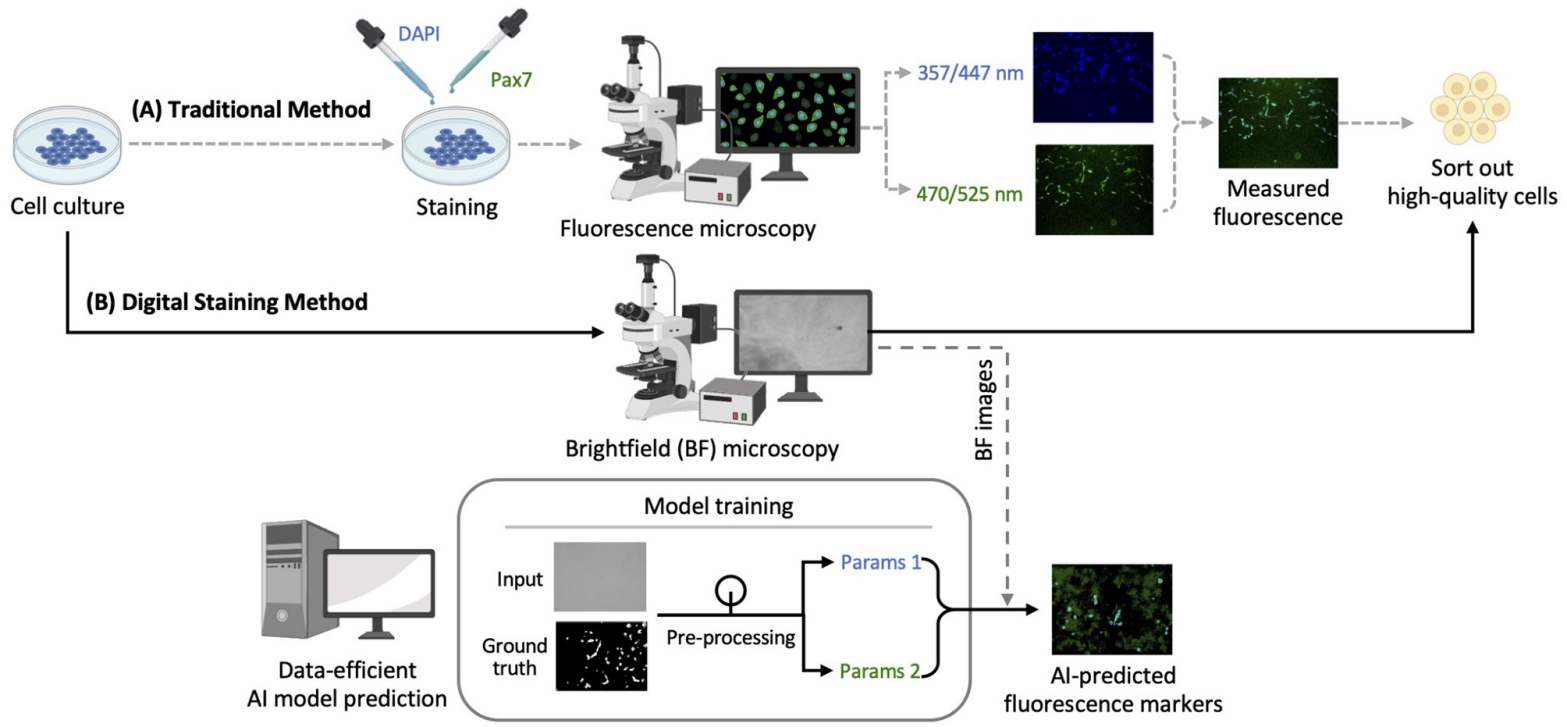
Schematic diagram for quality assessment of bovine cell culture: **(A)** Traditional immunofluorescence microscopy method; **(B)** digital staining method using deep learning for predicting fluorescent markers. Brightness and contrast of the example images were adjusted for publication clarity. BF, Brightfield.

As illustrated in Figure 1, image datasets were obtained using the traditional immunofluorescence microscopy method (Figure 1A). These datasets were used to train the CNN model architecture for digital staining, enabling the trained model to directly predict fluorescent markers in brightfield images without invasive sample preparation (Figure 1B). A set of brightfield (i.e., *input*) and fluorescence (i.e., *ground truth*) images were collected as detailed in Section 2.1. The technical details of AI prediction are provided in Section 2.2, including image preprocessing, model training and prediction, and post-processing.

### 2.1 Data collection

#### 2.1.1 Cell isolation and culture

BSCs were extracted from three-month-old Holstein bull calves (*n* = 3, body weight: 77.10 ± 2.02 kg) processed under USDA inspection at the Michigan State University Meat Laboratory. All procedures were approved by the MSU Institutional Animal Care and Use Committee (PROTO202000294), following the methods outlined in our previously published study (Kim et al., 2023). Following euthanization via a captive bolt, Longissimus muscle tissue was collected and transported in phosphate-buffered saline (PBS; Sigma Aldrich) with 3 × Antibiotic-Antimycotic (Thermo Fisher, Waltham, MA, USA). The muscle tissue was trimmed of vasculature, connective tissue, and fat, and ground using a sterile meat grinder. The tissue was enzymatically digested in 0.1% Pronase (Calbiochem, La Jolla, CA, USA) with Earl's Balanced Salt Solution (Sigma Aldrich, St. Louis, MO, USA) at 37 °C for 1 h in a shaking water bath. After centrifugation at room temperature at 1,500 × g for 4 min, the supernatant was discarded and the pellet resuspended in PBS. Cells were centrifuged at room temperature at 500 × g for 10 min, and this process was repeated to isolate a mononucleated cell pellet.

#### 2.1.2 DAPI and Pax7 staining

Cells were stained following our previously published method (Kim and Kim, 2023). Briefly, cells were seeded onto 4-well Lab-Tek chamber slides (Thermo Fisher) and incubated for 24 h at 38C in Dulbecco's Modified Eagle's Medium (Gibco, Waltham, MA, USA) supplemented with 10% fetal bovine serum (Thermo Fisher) and 1 × Antibiotic-Antimycotic under 95% O_2_/5% CO_2_. Cells were then fixed in 4% paraformaldehyde (Thermo Fisher) for 15 min at room temperature, washed with PBS, and permeabilized with 0.1% Triton X-100 (Thermo Fisher) in PBS for 15 min. Non-specific binding was blocked using a 2% bovine serum albumin (Thermo Fisher) in PBS for 1 h at 4 °C. Cells were incubated overnight at 4 °C with anti-Pax7 primary antibody (mouse monoclonal, 1:500, Developmental Studies Hybridoma Bank, Iowa City, IA, USA), followed by Alexa Fluor 488 anti-mouse IgG secondary antibody (1:1,000; Thermo Fisher) for 30 min at room temperature. After PBS washes, cells were counterstained with DAPI (1:1,000; Thermo Fisher) in PBS for 5 min at room temperature. Coverslips were mounted using Fluoromount-G Mounting Medium (Thermo Fisher) and sealed with nail polish.

#### 2.1.3 Brightfield and fluorescence microscopy

Slides were imaged using an EVOS M5000 inverted digital microscope (Thermo Fisher) at 20 × magnification in brightfield, DAPI fluorescence (excitation/emission: 357/447 nm), and green fluorescent protein (GFP) fluorescence (470/525 nm) modes. Imaging parameters were as follows: brightfield (18.88% light intensity, 16 ms exposure time, 1 dB gain); DAPI (7.553% intensity, 52.2 ms exposure, 30.6 dB gain); and Pax7 (43.75% intensity, 94.4 ms exposure time, 114 dB gain). Background fluorescence was recorded using blank chamber slides for DAPI and GFP channels. For each of the 48 biological replicates, triplicate sets of brightfield, DAPI, and Pax7 images were collected.

### 2.2 AI prediction of fluorescent markers

#### 2.2.1 Image preprocessing pipeline

**Figure 2:**
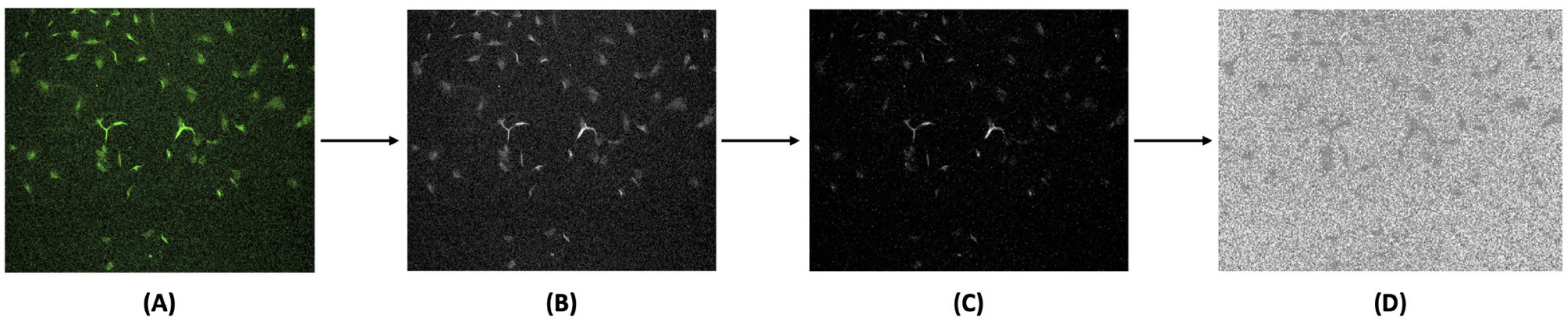
Image preprocessing pipeline for generating *target* fluorescent signals from *ground truth* fluorescence images. **(A)** Original *ground truth* data obtained by fluorescence microscopy were converted into **(B)** grayscale images, followed by **(C)** fluorescence denoising, and then **(D)** final normalization to enhance the *target* fluorescent signals for model training. Brightness and contrast of the example images were adjusted for publication clarity.

The collected *ground truth* data consisted of single-channel, colored fluorescence images. These images were pre-processed to generate *target* fluorescent signals, as shown in Figure 2. Since the CNN architecture used supports only grayscale images, the raw TIF images were first converted into grayscale. Next, background noise was reduced by subtracting the average of the background fluorescence scans during the fluorescence denoising step. This process helped remove random bright spots and normalize areas of high brightness, ensuring uniform identification of subcellular structures. Finally, PyTorch normalization transform was applied by scaling pixel intensities to zero mean and unit variance using statistics computed from the training set. Standard averaging techniques were ineffective due to the randomness of noise and the placement of subcellular components. The processed images were then split into *training* and *testing* datasets.

#### 2.2.2 Model training and prediction

The PyTorch-fnet framework originally developed for human cells by Ounkomol et al. (2018), is a CNN model based on the U-Net architecture for fluorescence prediction and was employed in this study. The U-Net variation used consists of three types of convolutional layers with varying kernel sizes and strides, each followed by batch normalization and a rectified linear unit (ReLU) activation function. While the original model architecture was used without modification, this study applies it to bovine satellite cell imaging, where structural heterogeneity and signal variability required a tailored preprocessing and post-processing pipeline to ensure reliable prediction. Built on the PyTorch library (Paszke et al., 2019), the fnet model supports both single-channel and multi-channel data and allows for flexible configuration of data transformations and evaluation metrics through JSON files. The *fnet_nn_2d* model architecture was selected, and training was performed using the Adam optimizer with a learning rate of 0.001. The loss function was a weighted mean squared error. A CSV file containing paths to the paired brightfield and fluorescence images for the *training* dataset was used as input. The model was trained to predict fluorescence images corresponding to DAPI and Pax7 staining using paired brightfield–fluorescence image sets. DAPI serves as a nuclear marker by binding to DNA, while Pax7 is a nuclear transcription factor that marks satellite cells. The model thus learns to infer the spatial distribution of these subcellular structures directly from transmitted light images, without explicit structural annotations. Following training, the model was applied to an unseen *testing* dataset. Prediction parameters were configured to match training conditions.

#### 2.2.3 Post-processing of model prediction results

To enhance the interpretability of the AI model predictions, post-processing steps were employed. While the original *ground truth* data were colored, the model was designed to use grayscale brightfield images as input and produce grayscale outputs, following the approach by Ounkomol et al. (2018). These model prediction outputs, initially in TIF format, were converted from grayscale to RGB and then to JPG format for visualization. Post-processing also involved color mapping, a standard digital image enhancement technique (Faridul et al., 2014). The original *ground truth* data were used to devise a color palette that maps the colorized output images closest to the original image selection. This step made the *predicted* images more consistent with traditional *ground truth* data and enhanced the visibility of subcellular components, particularly for noisy outputs.

The final step involved merging the output predictions for DAPI and Pax7 markers to produce the desired result of combined fluorescent markers. This was accomplished using scripts for image overlay and transparency adjustment, resulting in a more accurate prediction of the location and density of satellite cells. By merging the colorized predictions, we created a comprehensive visualization that resembles traditional multi-channel fluorescence microscopy.

#### 2.2.4 Model performance evaluation

Evaluating a model is essential for determining its effectiveness. However, establishing evaluation metrics or error values that accurately reflect model performance can be challenging. To address this, multiple standard statistical performance metrics were employed to assess model performance from various perspectives.

The Pearson *r* (correlation coefficient) (Nettleton, 2014), also used in the study by Ounkomol et al. (2018), measures the normalized covariance between the *target* and *predicted* images, with values ranging from –1 to 1. Values closer to 1 indicate higher correlation and image similarity. Mathematically, the absolute value of the Pearson *r* is given by:


$$\begin{array}{l} r=|\frac{\sum \left(x_{i}-\bar{x}\right)\left(y_{i}-ȳ\right)}{\sqrt{\sum {\left(x_{i}-\bar{x}\right)}^{2}\sum {\left(y_{i}-ȳ\right)}^{2}}}| \end{array}$$

where *x*_*i*_ and *y*_*i*_ are the individual data points, and $\bar{x}$ and ȳ are the respective means.

In addition, other widely used metrics such as the mean squared error (MSE) and the structural similarity index (SSIM) (Wang et al., 2004) were calculated. MSE, one of the most general measures of error, was computed by taking the average squared difference between the pixels of the *target* and *predicted* images. SSIM considers image texture and granularity, providing a more refined measure than simple MSE. Mathematically, the absolute value of SSIM is given by:


$$\begin{array}{l} SSIM\left(x,y\right)=|{\left[l\left(x,y\right)\right]}^{a}\times {\left[c\left(x,y\right)\right]}^{\beta }\times {\left[s\left(x,y\right)\right]}^{\gamma }| \end{array}$$

where


$$\begin{array}{lll} l\left(x,y\right) & = & \frac{2\mu_{x}\mu_{y}+C_{1}}{\mu_{x}^{2}+\mu_{y}^{2}+C_{1}}\\ c\left(x,y\right) & = & \frac{2\sigma_{x}\sigma_{y}+C_{2}}{\sigma_{x}^{2}+\sigma_{y}^{2}+C_{2}}\\ s\left(x,y\right) & = & \frac{\sigma_{xy}+C_{3}}{\sigma_{x}\sigma_{y}+C_{3}} \end{array}$$

Here, μ_*x*_ and μ_*y*_ are the pixel sample means, σ_*x*_ and σ_*y*_ are the standard deviations, σ_*xy*_ is the covariance, and *C*_1_, *C*_2_, and *C*_3_ are constants to stabilize the division with weak denominators.

To evaluate the significance of observed changes in model performance due to fluorescence denoising, a paired *t*-test was conducted for each metric (MSE, SSIM, and Pearson *r*) across all test samples. Metric values computed with and without denoising were compared for both DAPI and Pax7 predictions. All statistical tests were performed using the SciPy library, with a significance threshold of *p* < 0.05.

## 3 Results and discussion

### 3.1 Evaluation of model performance with enhanced visual interpretability

To evaluate model performance, the *predicted* fluorescence images were qualitatively compared to the *target* fluorescent signals. The use of post-processing techniques, including color mapping and image overlay, facilitated a clearer interpretation of the fluorescent signals. These techniques provided vital contextual information, enhancing the perceptual quality of the predictions. The merged predictions of DAPI and Pax7 markers enabled precise localization of BSCs on input brightfield images, demonstrating the model's capability in digital staining for cell culture quality assessment. Because DAPI binds strongly to DNA, its fluorescence signal corresponds to nuclear localization and overall cell density. Pax7 is a transcription factor expressed in the nuclei of satellite cells, making its fluorescence signal a marker of satellite cell identity and differentiation status. The model learns to approximate these subcellular distributions based on structural features observed in brightfield images.

#### 3.1.1 DAPI predictions exhibit better performance compared to Pax7

**Figure 3:**
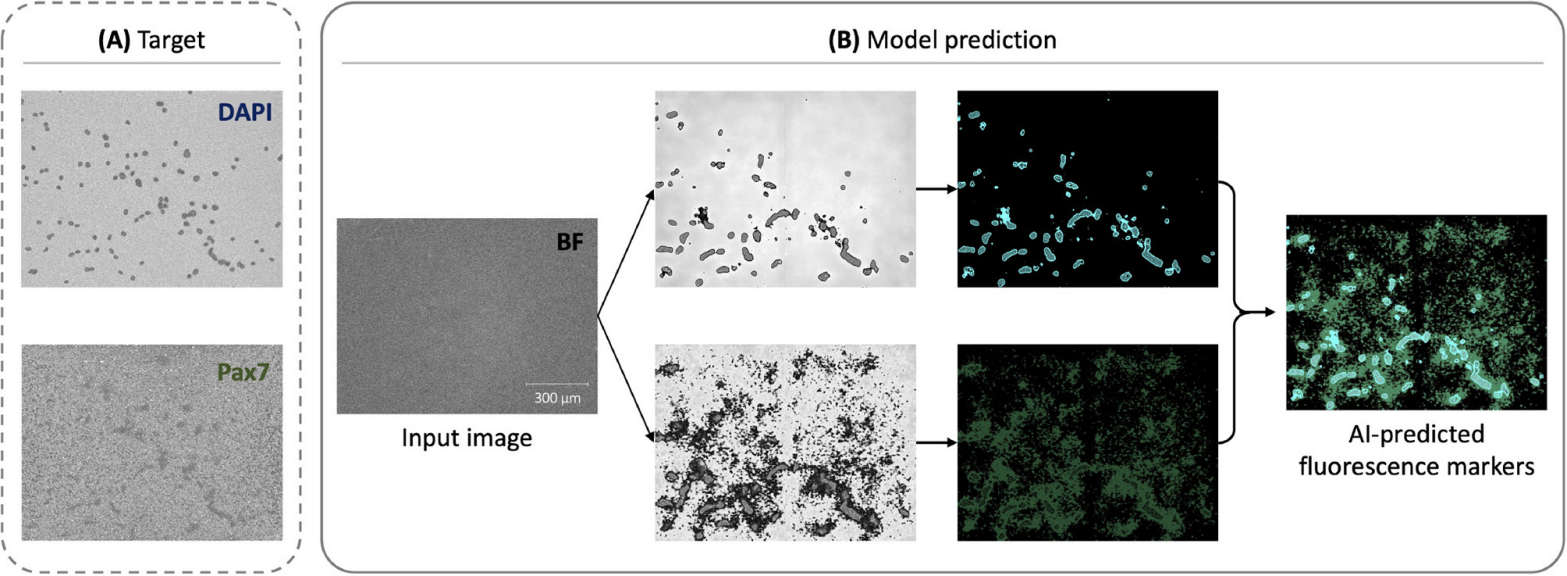
Representative examples of AI-enabled prediction of multiple fluorescent markers from a single BF image: **(A)** Preprocessed *target* fluorescence signals for DAPI (top) and Pax7 (bottom), which were measured experimentally and used as labels during training. These were generated from *ground truth* fluorescence microscopy images through the preprocessing pipeline shown in Figure 2 and used as training labels, but included in model prediction. **(B)** Corresponding model predictions for DAPI and Pax7, shown with post-processing and overlaid as a composite for qualitative interpretation.

As shown in Figure 3, the model predictions for DAPI achieved better performance compared to Pax7. The DAPI predictions displayed less background noise and variability, attributed to the uniform staining and distribution of DAPI, which binds to DNA. In contrast, Pax7 predictions were more variable due to the inconsistent expression and localization of Pax7 in cells. This observation suggests that the fnet model architecture is particularly well-suited for predicting DAPI fluorescence, aligning with its original design for subcellular structures like DNA and cell membranes (Ounkomol et al., 2018).

#### 3.1.2 Biological implications in improving model performance

The use of DAPI and Pax7 in this study was intended to assess the proliferation and differentiation capabilities of BSCs. These fluorophores target specific cellular components, enhancing contrast and resolution. However, biological samples often exhibit noisy backgrounds and diffuse signals, particularly with Pax7, due to the heterogeneity in myogenic differentiation of BSCs (Kong et al., 2023). This variability poses significant challenges for signal quantification and automated analysis.

Deep learning techniques rely heavily on high-quality data and tend to underperform when such data are scarce. This issue is particularly relevant in predicting immunofluorescent signals like Pax7, where the limited availability of labeled data exacerbates the challenge. Rather than training a model from scratch, fine-tuning a pre-trained model with local data has been shown to be a more effective strategy (Tajbakhsh et al., 2016; Moen et al., 2019). Thus, future studies should focus on improving pre-training strategies specifically for Pax7 with heterogeneous biological states. This could involve using attention-based networks to segment subcellular components with varying health states (Wang et al., 2023), or incorporating deep learning-based identification of cell differentiation (Zhu et al., 2021). Enhancing the handling of Pax7 signals is crucial for advancing the reliability of deep learning models in predicting these markers.

### 3.2 Improved consistency of predictions through fluorescence denoising

**Figure 4:**
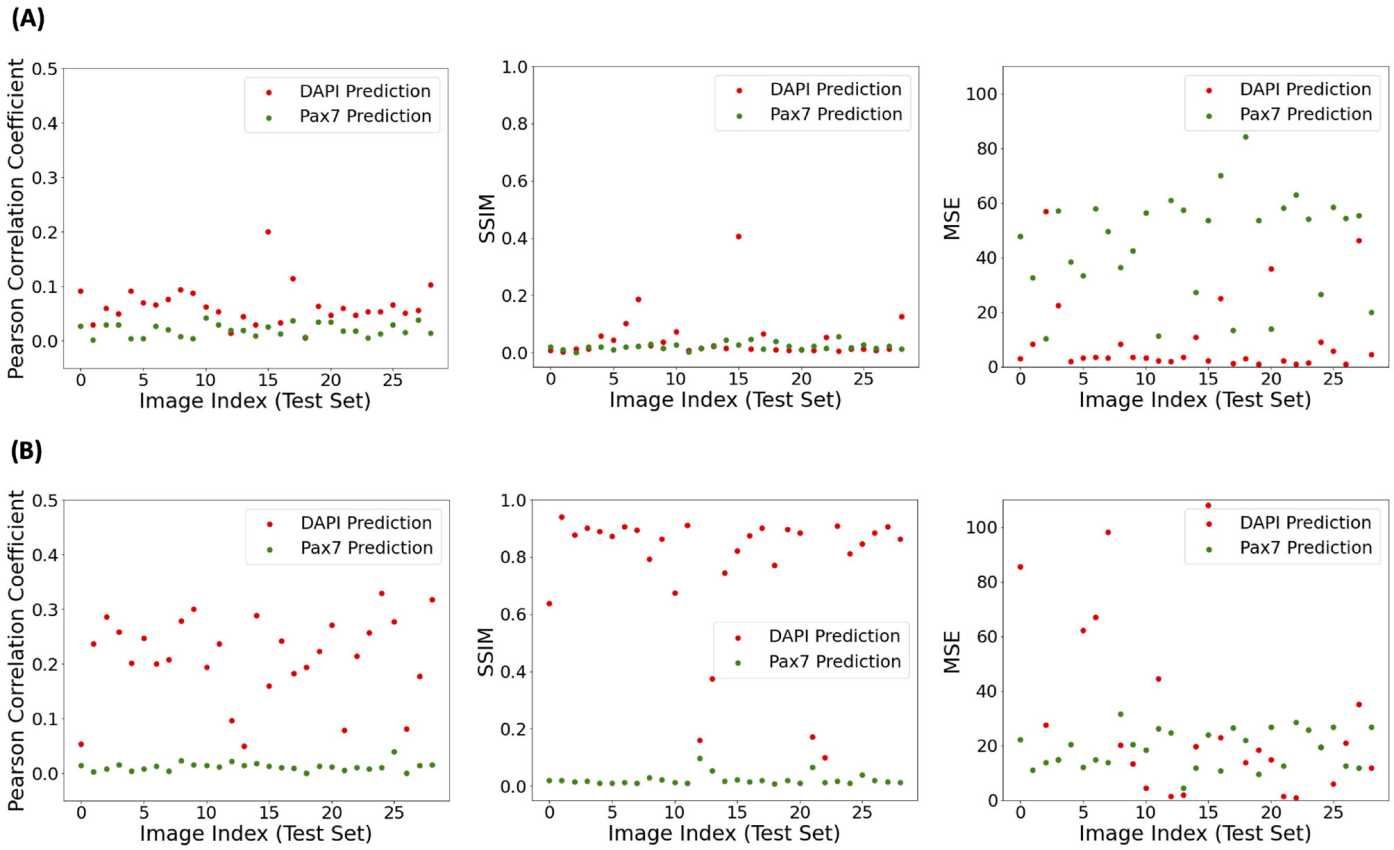
Statistical performance evaluation of AI-predicted DAPI and Pax7 fluorescence signals from BF images using models trained on different *targets*. **(A)** Model trained using unprocessed *ground truth* fluorescence images. **(B)** Model trained using preprocessed fluorescence signals (as in Figure 2). Metrics shown from left to right are Pearson correlation coefficient (higher indicates better correlation), structural similarity index (SSIM; higher indicates greater similarity), and mean squared error (MSE; lower indicates better accuracy) between predicted and *ground truth* images.

In addition to the individual visual assessment of model performance, its consistency was investigated using selected statistical performance evaluation metrics: Pearson *r*, SSIM, and MSE. The top row of Figure 4 illustrates the performance of the model trained on *target* fluorescent signals derived from raw data without fluorescence denoising. All three metrics showed similar trends for both DAPI and Pax7 predictions, with slightly higher values for DAPI predictions in the Pearson *r*. The average Pearson *r* for DAPI was 0.065, SSIM was 0.047, and MSE was 9.507. For Pax7, the average Pearson *r* was 0.020, SSIM was 0.022, and MSE was 44.753. The bottom row Figure 4 shows the changes in these evaluation metric values after applying our image preprocessing pipeline for fluorescence denoising, as depicted in Figure 2. This preprocessing resulted in higher values for SSIM and Pearson *r* metrics, indicating an overall improvement in model performance. For MSE, we got lower values for Pax7 indicating an improvement, but values for DAPI increased. After denoising, the average Pearson *r* for DAPI increased to 0.212, SSIM to 0.761, and MSE to 41.571. For Pax7, the average Pearson *r* increased to 0.124, SSIM to 0.023, while MSE decreased to 18.793.

#### 3.2.1 Feasibility of evaluation metrics in digital staining

As shown in Figure 4, MSE values increased with fluorescence denoising in our image preprocessing pipeline, suggesting greater errors in pixel-wise predictions. Despite this, visual assessment of the final outputs showed improved model performance with our image preprocessing. This improvement is consistent with the increase in the Pearson *r* and SSIM values, indicating better correlation and structural similarity between the *target* and *predicted* signals. This discrepancy suggests that MSE may not be the most appropriate metric for evaluating model performance in this context. While MSE measures pixel-wise accuracy, it may not fully capture perceptual quality, spatial context, or signal-to-noise ratio. Perceptual quality, relating to human visual perception, is better captured by metrics like SSIM that consider structural information (Wang et al., 2004). SSIM evaluates luminance, contrast, and structure, making it more sensitive to visual perception than MSE. Spatial context is crucial in biological imaging, where the arrangement and relationship of cellular structures matter more than exact pixel values. SSIM captures spatial information and provides a better understanding of image quality (Wang and Bovik, 2002). Furthermore, the signal-to-noise ratio is critical in microscopy images, where high background noise can obscure meaningful signals. MSE does not account for noise distribution, whereas SSIM can provide a more nuanced assessment of image quality by considering noise levels and their impact on structural similarity (Brunet et al., 2012). Overall, while MSE measures pixel-wise accuracy, it falls short in capturing the perceptual quality, spatial context, and signal-to-noise ratio essential for evaluating digital staining in cell microscopy.

Additionally, the Pearson *r* measures the linear relationship between *target* and *predicted* signals, providing insights into overall trend alignment rather than pixel-wise accuracy. The average Pearson *r* obtained in this study was lower than in the original study of the fnet model (Ounkomol et al., 2018), where the value for DNA was over 0.6. This discrepancy can be attributed to differences in the input data. The previous study used 3D z-stacks of brightfield images or 2D electron micrographs, which provide more comprehensive information about subcellular structures and thus achieved higher correlation values. In contrast, our study used only single focal plane data, which may lack some spatial context. Despite this, our model still performed reasonably well, demonstrating the robustness of our approach in predicting fluorescent signals from 2D brightfield data. This adaptation underscores the practical applicability of deep learning in image-based quality assessment of BSC culture, enabling cost-effective digital staining in cell imaging.

#### 3.2.2 Statistical validation of metric improvements

To assess whether the observed differences in evaluation metrics before and after fluorescence denoising were statistically meaningful, a paired *t*-test was performed on the metric values across all test images. The *p*-values indicate whether the observed differences are statistically significant, and the corresponding *t*-values reflect the direction and magnitude of change in each metric. As shown in Table 1, the *p*-values indicate statistically significant differences for most tests, except the SSIM metric for Pax7. For DAPI, the *t*-values indicate that both SSIM and Pearson *r* increased with denoising, thereby improving prediction. While the average SSIM and Pearson *r* for Pax7 also increased slightly, these changes were not statistically significant, in line with the findings discussed in Section 3.1.1. The MSE metric showed inconsistent changes across replicates, with differing signs in the t-values for DAPI and Pax7, further supporting the limitations of using MSE in this context, as discussed in Section 3.2.1.

**Table 1:** Paired *t*-test results across the three different evaluation metrics with and without denoising.

**Marker**	**MSE**		**SSIM**		**Pearson** ***r***	
	***p*-value**	***t*-value**	***p*-value**	***t*-value**	***p*-value**	***t*-value**
DAPI	9.76 × 10^−4^	–3.68	2.59 × 10^−15^	–15.56	5.76 × 10^−10^	–9.21
Pax7	5.52 × 10^−7^	6.45	0.829	–0.218	1.73 × 10^−3^	3.47

MSE, Mean squared error; SSIM, Structural similarity index measure; Pearson *r*, Pearson correlation coefficient; *p*-value, probability under the null hypothesis; *t*-value, test statistic from paired *t*-test.

#### 3.2.3 Importance of preprocessing in addressing biological heterogeneity

To effectively utilize existing models and fine-tune them to specific datasets, data preprocessing is essential, especially in managing inconsistent data quality and mitigating the risk of overfitting. In our approach to fluorescence denoising, background fluorescence scans were subtracted from the raw data. Standard normalization techniques were ineffective due to randomly scattered noise, which often removed the actual areas of interest. This noisy fluorescence background in the raw data necessitated optimization of brightness parameters, such as light intensity, exposure time, and gain for each fluorescence channel, as described in Section 2.1.3, resulting in inadvertently elevated non-specific background fluorescence. To address this issue, a fluorescence denoising technique for each channel was implemented in our preprocessing pipeline (Figure 2), which substantially improved the consistency of model predictions, as demonstrated in Figure 4. This adjustment enhances the reliability of results by accommodating variability in brightness parameters across different fluorescence channels. Moreover, researchers have explored various experimental approaches to improve staining methods and reduce non-specific binding (Zaqout et al., 2020). Additionally, algorithms have been developed to digitally remove autofluorescent signals (Wang et al., 2022). These efforts underscore the ongoing need to improve fluorescence specificity in the quantitative assessment of microscopy images. Continued research is essential to enhance the quality of *training* data, thereby advancing the application of deep learning for precise fluorescent marker prediction in cell imaging. Beyond improving prediction consistency in this study, the tailored preprocessing and post-processing visualization pipeline offers a generalizable strategy for adapting deep learning architectures to biologically complex systems. Although the original U-Net-based fnet model was developed for standardized cell types and imaging conditions (Ounkomol et al., 2018), applying it directly to primary bovine satellite cells, which exhibit structural variability and high background noise, highlighted the need for such pipeline-level adjustments. These findings demonstrate that careful data handling can extend the applicability of existing models to new biological domains, enabling more robust and interpretable predictions in less controlled settings.

### 3.3 Limitations and future directions

While this study demonstrated the feasibility of predicting fluorescent markers from brightfield images using deep learning, several limitations should be acknowledged. Although the dataset included triplicate images per biological replicate (totaling 144 images), the number of unique biological samples was limited to 48. This restricts the range of biological and experimental variability represented in the training set, which may affect model generalizability to new datasets. Additionally, while we focused on two biologically relevant markers (DAPI and Pax7) commonly used in muscle tissue analysis, further validation using additional fluorescent markers and imaging conditions is necessary to assess the broader utility of this approach. Given the variability observed in Pax7 predictions, expanding this method to other markers, including those capturing different stages of myogenic differentiation or derived from different cell types, will be important for evaluating generalizability and robustness. Future work should also consider transfer learning or attention-based architectures to better accommodate signal heterogeneity across marker types (Tajbakhsh et al., 2016; Papanastasiou et al., 2024). Moreover, although we evaluated prediction performance using biological replicates and standard image similarity metrics, future studies should incorporate ablation analyses to isolate the contributions of preprocessing steps, architectural components, or specific markers to model performance.

## 4 Conclusions

In summary, our study presents a non-invasive method for assessing BSC cultures using deep learning to predict multiple fluorescent signals from a single brightfield image. Using DAPI and Pax7 as biomarkers and employing a CNN model based on U-Net with an optimized preprocessing pipeline, we achieved substantial improvements in prediction performance and consistency. Evaluation using the Pearson *r* and SSIM demonstrated that these metrics capture perceptual quality and spatial context more effectively than pixel-wise error measurements. Enhanced visualization techniques further increased the interpretability of the predicted signals. These findings highlight the critical role of data preprocessing and demonstrate the potential of AI-driven non-invasive methods for cellular quality assessment in tissue-engineered muscle constructs. Our approach offers promising prospects for integrating advanced machine learning techniques in cell biology applications and improving resource management in agricultural and biotechnological systems.

## Data Availability

The code is available at https://github.com/food-ai-engineering-lab/bsc-fluorescence-prediction. Future updates will be integrated to this repository.

## References

[B1] Bilodeau A. Delmas C. V. L. Parent M. De Koninck P. Durand A. Lavoie-Cardinal F. (2022). Microscopy analysis neural network to solve detection, enumeration and segmentation from image-level annotations. Nat. Mach. Intell. 4, 455–466. 10.1038/s42256-022-00472-w

[B2] Binder A. Bockmayr M. Hgele M. Wienert S. Heim D. Hellweg K.. (2021). Morphological and molecular breast cancer profiling through explainable machine learning. Nat. Mach. Intell. 3, 355–366. 10.1038/s42256-021-00303-4

[B3] Brunet D. Vrscay E. R. Wang Z. (2012). On the mathematical properties of the structural similarity index. IEEE Trans. Image Proc. 21, 1488–1499. 10.1109/TIP.2011.2173206 22042163

[B4] Cheng S. Fu S. Kim Y. M. Song W. Li Y. Xue Y.. (2021). Single-cell cytometry via multiplexed fluorescence prediction by label-free reflectance microscopy. Sci. Adv. 7:eabe0431. 10.1126/sciadv.abe0431 33523908 PMC7810377

[B5] Cho K. Choi E.-S. Kim J.-H. Son J.-W. Kim E. (2022). Numerical learning of deep features from drug-exposed cell images to calculate IC50 without staining. Sci. Rep. 12:6610. 10.1038/s41598-022-10643-9 35459284 PMC9033873

[B6] Christiansen E. M. Yang S. J. Ando D. M. Javaherian A. Skibinski G. Lipnick S.. (2018). *In silico* labeling: predicting fluorescent labels in unlabeled images. Cell 173, 792–803.e19. 10.1016/j.cell.2018.03.040 29656897 PMC6309178

[B7] Ding S. Swennen G. N. M. Messmer T. Gagliardi M. Molin D. G. M. Li C.. (2018). Maintaining bovine satellite cells stemness through p38 pathway. Sci. Rep. 8:10808. 10.1038/s41598-018-28746-7 30018348 PMC6050236

[B8] Faridul H. S. Pouli T. Chamaret C. Stauder J. Trémeau A. Reinhard E. (2014). “A survey of color mapping and its applications,” in Eurographics - *State of the Art Reports*, eds. S. Lefebvre, and M. Spagnuolo (Strasbourg, France: The Eurographics Association), 43–67.

[B9] Kim W. S. Daddam J. R. Keng B. H. Kim J. Kim J. (2023). Heat shock protein 27 regulates myogenic and self-renewal potential of bovine satellite cells under heat stress. J. Animal Sci. 101:skad303. 10.1093/jas/skad303 37688555 PMC10629447

[B10] Kim W. S. Kim J. (2023). Exploring the impact of temporal heat stress on skeletal muscle hypertrophy in bovine myocytes. J. Therm. Biol. 117:103684. 10.1016/j.jtherbio.2023.103684 37625343

[B11] Kong Y. Ao J. Chen Q. Su W. Zhao Y. Fei Y.. (2023). Evaluating differentiation status of mesenchymal stem cells by label-free microscopy system and machine learning. Cells 12:1524. 10.3390/cells12111524 37296645 PMC10252613

[B12] Kromp F. Fischer L. Bozsaky E. Ambros I. M. Dorr W. Beiske K.. (2021). Evaluation of deep learning architectures for complex immunofluorescence nuclear image segmentation. IEEE Trans. Med. Imaging 40, 1934–1949. 10.1109/TMI.2021.3069558 33784615

[B13] Lee S. Y. Kang H. J. Lee D. Y. Kang J. H. Ramani S. Park S.. (2021). Principal protocols for the processing of cultured meat. J. Animal Sci. Technol. 63, 673–680. 10.5187/jast.2021.e40 34447947 PMC8367396

[B14] Messmer T. Klevernic I. Furquim C. Ovchinnikova E. Dogan A. Cruz H.. (2022). A serum-free media formulation for cultured meat production supports bovine satellite cell differentiation in the absence of serum starvation. Nat. Food 3, 74–85. 10.1038/s43016-021-00419-1 37118488

[B15] Moen E. Bannon D. Kudo T. Graf W. Covert M. Van Valen D. (2019). Deep learning for cellular image analysis. Nat. Methods 16, 1233–1246. 10.1038/s41592-019-0403-1 31133758 PMC8759575

[B16] Nettleton D. (2014). “Chapter 6 - selection of variables and factor derivation,” in Commercial Data Mining, ed. D. Nettleton (Boston: Morgan Kaufmann), 79–104. 10.1016/B978-0-12-416602-8.00006-620641233

[B17] Ounkomol C. Seshamani S. Maleckar M. M. Collman F. Johnson G. R. (2018). Label-free prediction of three-dimensional fluorescence images from transmitted-light microscopy. Nat. Methods 15, 917–920. 10.1038/s41592-018-0111-2 30224672 PMC6212323

[B18] Papanastasiou G. Dikaios N. Huang J. Wang C. Yang G. (2024). Is attention all you need in medical image analysis? A review. IEEE J. Biomed. Health Inf. 28, 1398–1411. 10.1109/JBHI.2023.3348436 38157463

[B19] Paszke A. Gross S. Massa F. Lerer A. Bradbury J. Chanan G.. (2019). “Pytorch: an imperative style, high-performance deep learning library,” in Advances in Neural Information Processing Systems, eds. H. Wallach, H. Larochelle, A. Beygelzimer, F. d' Alch-Buc, E. Fox, and R. Garnett (Curran Associates, Inc.).

[B20] Ronneberger O. Fischer P. Brox T. (2015). “U-net: convolutional networks for biomedical image segmentation,” in Medical Image Computing and Computer-Assisted Intervention MICCAI 2015, eds. N. Navab, J. Hornegger, W. M. Wells, and A. F. Frangi (Cham: Springer International Publishing), 234–241. 10.1007/978-3-319-24574-4_28

[B21] Samek W. Wiegand T. Müller K.-R. (2017). Explainable artificial intelligence: understanding, visualizing and interpreting deep learning models. arXiv preprint arXiv:1708.08296.

[B22] Seale P. Sabourin L. A. Girgis-Gabardo A. Mansouri A. Gruss P. Rudnicki M. A. (2000). Pax7 is required for the specification of myogenic satellite cells. Cell 102, 777–786. 10.1016/S0092-8674(00)00066-0 11030621

[B23] Stout A. J. Arnett M. J. Chai K. Guo T. Liao L. Mirliani A. B.. (2023). Immortalized bovine satellite cells for cultured meat applications. ACS Synth. Biol. 12, 1567–1573. 10.1021/acssynbio.3c00216 37146268

[B24] Tajbakhsh N. Shin J. Y. Gurudu S. R. Hurst R. T. Kendall C. B. Gotway M. B.. (2016). Convolutional neural networks for medical image analysis: full training or fine tuning? IEEE Trans. Med. Imaging 35, 1299–1312. 10.1109/TMI.2016.2535302 26978662

[B25] von Maltzahn J. Jones A. E. Parks R. J. Rudnicki M. A. (2013). Pax7 is critical for the normal function of satellite cells in adult skeletal muscle. Proc. Nat. Acad. Sci. 110, 16474–16479. 10.1073/pnas.1307680110 24065826 PMC3799311

[B26] Wang R. Butt D. Cross S. Verkade P. Achim A. (2023). Bright-field to fluorescence microscopy image translation for cell nuclei health quantification. Biol. Imag. 3:e12. 10.1017/S2633903X23000120 38510164 PMC10951917

[B27] Wang Y. X. Holbrook C. A. Hamilton J. N. Garoussian J. Afshar M. Su S.. (2022). A single cell spatial temporal atlas of skeletal muscle reveals cellular neighborhoods that orchestrate regeneration and become disrupted in aging. bioRxiv preprint 2022.06.10.494732. 10.1101/2022.06.10.494732

[B28] Wang Z. Bovik A. (2002). A universal image quality index. IEEE Signal Process. Lett. 9, 81–84. 10.1109/97.995823

[B29] Wang Z. Bovik A. Sheikh H. Simoncelli E. (2004). Image quality assessment: from error visibility to structural similarity. IEEE Trans. Image Proc. 13, 600–612. 10.1109/TIP.2003.819861 15376593

[B30] Zaqout S. Becker L.-L. Kaindl A. M. (2020). Immunofluorescence staining of paraffin sections step by step. Front. Neuroanat. 14:582218. 10.3389/fnana.2020.582218 33240048 PMC7680859

[B31] Zhu Y. Huang R. Wu Z. Song S. Cheng L. Zhu R. (2021). Deep learning-based predictive identification of neural stem cell differentiation. Nat. Commun. 12:2614. 10.1038/s41467-021-22758-0 33972525 PMC8110743

